# Comparative Law and Christianity—A Plank in the Eye?

**DOI:** 10.1093/ojls/gqad021

**Published:** 2023-10-05

**Authors:** Jaakko Husa

**Keywords:** comparative law, legal families, religious law, Christianity, epistemology

## Abstract

This article examines the epistemic bias of comparative law scholarship. Comparatists are unable or unwilling to recognise the religious dimensions in Western law as they see religion only in the context of non-Western law. This problem is typical of modern macro-comparative law, which fails to recount the influence of Christianity on Western law and legal culture. The article invites legal scholars to reach beyond the notions of ‘religious law’ and ‘secular law’ in terms of classifying the world’s legal systems. Firstly, the article explains how comparative law has a problematic relationship with religion; secondly, it shows that, despite Christianity having been deemed a thing of the past, its influence can and should also be charted in modern law. I argue for a need to rethink the manner in which Western law is depicted as a thoroughly secular law as opposed to the religious law of exoticised others.

Why do you look at the speck in your brother’s eye, but fail to notice the beam in your own eye? How can you say to your brother, ‘Let me take the speck out of your eye,’ while there is still a plank in your own eye?^1^

– Matthew 7:3

## 1. Introduction

As a field of legal study, comparative law has had its fads and follies, but some things seem to form a permanent part of the intellectual fabric of comparative law scholarship. Comparative law scholars are accustomed to relying on an established conceptual framework when they describe foreign legal systems. Such well-known concepts as legal family, legal culture and legal tradition are constantly referred to. Indeed, it is virtually impossible to read comparative law scholarship without repeatedly stumbling upon such notions as civil law and common law. Reflective discussion on these macro-concepts is not, however, widespread practice. Many sorts of legal scholars refer routinely to legal families without unpacking what such concepts as common law, civil law or Islamic law contain. Unpacking, if done at all, is left for comparative law scholars.^2^

Beyond comparative law theory, discussion rarely focuses on how comparatists attach labels to legal systems when they explain why a certain system belongs under a certain macro-concept. Regardless, it is clear that comparative law scholars typically apply certain criteria when they classify the world’s legal systems.^3^ One criterion for grouping systems is religion, although the role of religion is paradoxical as it is simultaneously applied but not applied. Religion is bafflingly visible yet invisible. In general, it can be argued that religion suffers a more or less marginalised existence in modern comparative law.^4^ Christianity, in particular, is deemed to be in the past, and is thus something for legal historians to discuss. Thick volumes on the subject mention religion in the context of Western law only in passing.^5^

My argument is that there seems to be a kind of an epistemic allergy in terms of Western law and religion, whereas non-Western law and religion are seen in connection almost instinctively.^6^ Scholarship in comparative law and religion has not produced much by way of published articles and books.^7^ And whereas civil law, common law and systems that mix the two (mixed systems) are considered as professional and non-religious, many other systems are termed religious, or at least are seen as having a much closer relation to religion than Western professionalised secular law.^8^ The problem with this scholarly practice is that it fails to recognise the role of religion in Western systems. Berman points to the failure of most comparatists ‘to explore the influence of Christianity on various Western families of law’.^9^ It will be argued here that this is because of epistemic blindness.^10^ This blindness is one-sided in the sense that comparatists are capable of attaching religious aspects to legal systems that exist beyond the boundaries of Western law. The epistemic reach of comparative law scholarship seems to limit its vision; self-reflection is apparently missing.

The situation recalls Christian teaching on hypocritical or selfish criticism of others that the introductory biblical quote expresses. In this tenet, the idea is that human beings do not have the authority to judge the righteousness of others by imposing their own preferences as the standard. Essentially, the epistemic idea is that because we ourselves are not blameless, we are in no position to act as judges. In short, the teaching is that we humans tend to be blind to our own shortcomings. It is argued here that comparatists suffer a similar kind of epistemic blindness. If Whitman is right when he says that comparative law and religion has changed from an afterthought into ‘a topic of burning importance’, then the epistemic bias is problematic.^11^ The objective of this article is to invite legal scholars to reach beyond the seemingly neutral notions of ‘religious law’ and ‘secular law’ against the backdrop of classifications of the world’s legal systems. Underplaying the significance of religion for Western law seems to be an issue for the comparative study of law in general. Notwithstanding, this article addresses the issue from the viewpoint of macro-comparative law.^12^

The macro-comparative study of law focuses on legal systems or legal cultures. This kind of comparative law typically focuses on methods of legislating, the style of legal documents, fields of law or doctrine on legal sources. Nevertheless, the difference between micro- and macro-comparison is flexible as to its nature.^13^ How does one bring in study of religion here? When the relationship between law and religion is addressed, theology and law come close to each other, leading, for instance, to such an amalgamated field of study as legal theology.^14^ The approach in this article is not legal-theological, but remains within comparative law scholarship.^15^ The argument here does not concern comparative religious law, but the manner in which comparative law deals with religion. To that end, Christianity is seen through comparative law scholarship, not through theology.^16^

Applying the comparative law viewpoint means that this article does not as such discuss Christian theology, which may—as is not denied here—offer critical capacity to the world of law.^17^ Here the focus is on how comparative law theorists have dealt with religious themes and law. This means that this article falls outside the more common types of comparative law and religion scholarship, which can be divided into comparison of systems with different religious traditions, comparison of religiously relevant rights or a broad socio-legal comparison based on the notion of legal culture.^18^ Of these research areas, rights-based comparison has been the most popular, with issues such as separation of church and state and individual belief and manifestation having been addressed under the umbrella of comparative human rights law.^19^ When Christianity and law is taken into account comparatively, it tends to concern only canon law.^20^ Yet it can be said that the role of canon law in today’s comparative law is only marginal.^21^ Here the focus is on the epistemic scope of comparative law in the context of Western law.

This article proceeds as follows. Section 2 explains what macro-comparative law scholarship is about and why it has a problematic relationship with religion. Section 3 discusses how Christianity has been deemed a thing of the past in Western law even though this is not the case if law is studied beneath its secular surface. Section 4 then argues that comparative law should also detect the effect of religion on Western law. Section 5 concludes by elucidating why it makes sense in terms of the study of comparative law to distinguish underlying subtleties in Western law that challenge the assumption that it is secular.

## 2. Macro-comparative Classifications and Religion

The key activity of macro-comparative law scholarship has been to map, in one way or another, the world’s legal systems. Global mapping is not unique to comparative law as it is the equivalent of classifying religions into indigenous religions, Judaism, Christianity, Islam, Hinduism, Buddhism and Confucianism.^22^ Indeed, classifying languages into different language families (eg Indo-European, Afro-Asiatic and Sino-Tibetan) also comes close to macro-comparative law.^23^ The goal of these kinds of taxonomies is to organise a certain field of study on a macro-level in order to offer an overall view but without providing an excessive amount of detail. These classifications are very broad generalisations, as conceiving diverse indigenous religions as a single group clearly demonstrates. It goes without saying that this casual grouping is deeply problematic as its deep roots reach down to 19th- and 20th-century colonial scholarship and perspectives.

Much of the 20th-century comparative law scholarship concerned classifying legal systems under different macro-concepts. Of these, civil law and common law are the most widely used and best known. The role of religion has been somewhat curious in these classifications.^24^ The reader can sense that the religious dimension lies mostly somewhere beneath the surface, even though it is not openly discussed except in connection with non-Western law or with Western law of the past. Macro-comparative law differs from the normal coupling of comparative law research and religion as there is no attempt to find commonalities or shared values.^25^ In any case, it seems evident that religion(s) emerge only in macro-comparison because visible religious influence has dropped out of sight from most micro-comparative law scholarship.^26^ Arguably, Western comparative law scholars have simply neglected elements of law that transcend secular rationality and place their *own* law in contact with religion.^27^ While modern Western comparatists make room especially for Islamic law, other forms of religious law are seen as ‘much less significant in our times’ and end up being discussed in the form of an excursus or as a part of secularised law.^28^ Secularism, however, is not a neutral category in the conceptual toolbox of the comparatist because it is an ideology and array of practices that are more consistent with Christian traditions than, for example, with Jewish or Islamic ones.^29^

Classifications and taxonomies of legal systems have been a feature of legal scholarship for a long time. They became commonplace in the late 19th and early 20th century, yet even more recent comparison of legal cultures has not changed the approach of leaving religion in the shadows of Western law, undiscussed or receiving minimal attention.^30^ Comparing religious laws and placing Christianity as an object of study seldom occurs.^31^ From this viewpoint, Reimann’s argument according to which ‘we have arrived at a much richer, especially more nuanced and dynamic, view of the world map of law than we had in 1950’ seems flawed if we take into account the dimension of Christianity and Western law.^32^ Instead of becoming more nuanced, religion has all but faded out. This dwindling is problematic when Western law is juxtaposed with non-Western law and tacitly depicted as a thoroughly secularised form of law.

### A. The Old School and Its Variants

It has been argued that classic writings on comparative law barely mention a relationship between religion and law in different cultures.^33^ On closer inspection, however, mainstream comparative law scholarship of the 20th century was not blind in terms of the relationship between law and religion. The problem lies in the manner in which religion is taken into account. Firstly, the role of religions has been limited to non-Western law. Secondly, the focus has been so heavily on non-religious—secular—law that the amount of attention devoted to law deemed religious is minimal. The best-known representative of this approach is the influential mainstream volume by Zweigert and Kötz, which has been translated into several languages.^34^ This book can be described as a paradigmatic example of comparative law scholarship of the late 20th century. The first editions, which came out in German came out in the late 1960s and early 1970s; numerous editions have had an impact on generations of legal scholars around the world.

The thick volume of Zweigert and Kötz’s 1998 edition touches on religion in 16 pages, dealing with Islamic law in eleven pages and Hindu law in six.^35^ Discussions on the Romanistic (French) legal family, the Germanic legal family, the Anglo-American legal family, the Nordic legal family and law in the far east (China, Japan) are dealt with in 228 pages.^36^ Religion is also briefly discussed in the context of reception of the French Code civil in Africa as ‘the natives had their own courts, staffed by tribal dignitaries, Islamic judges (cadis)’ and French colonial officials and judges.^37^ According to Zweigert and Kötz’s view, Western law apparently had nothing to do with religion—or, if it had anything at all to do with it, it was in the distant past and bore no contemporary relevance. In a way, it is hardly surprising that during the era in which their volume was penned it was easy to think that ‘religion was steadily fading in significance’.^38^ Curiously, the predecessors of Zweigert and Kötz were not timid two decades earlier when it came to drawing the connection between Western law and Christianity.^39^ One can only speculate that the change is connected to the more general secularisation of Western societies from the 1960s onwards.^40^

Whereas Zweigert and Kötz come from German legal academia, French legal academia has also produced considerable scholarship on macro-comparative law, continuing along the path pioneered by David’s influential book *Les grands systèmes de droit contemporains*.^41^ For instance, Cuniberti’s book on legal families, or *grands systèmes de droit*, continues along the lines of French macro-comparative law. His book takes religious law more broadly into account than do Zweigert and Kötz as it offers far more space to religion and law.^42^ The book has three parts in which religious law is addressed. The most important part is the chapter on Islamic law, which discusses the history of Islamic law, classical Islamic law and modern applications of the law against the backdrop of examples.^43^ Two other parts deal with Hindu law as part of Indian law^44^ and the sacred in the context of African traditional law.^45^ Like Zweigert and Kötz, Cuniberti does not deal with religion in the context of Western law—religious awareness is for others.

A different kind of classification taking religion into account was proposed by Mattei in 1997.^46^ Instead of relying on classic legal families, he sought to identify hegemonic cultural patterns of legal systems. In a Weberian ideal-type manner, Mattei distinguished three basic types of legal system, namely professional law, political law and traditional law. In essence, professional law contained what can be labelled as Western law, including civil law, common law and systems that combine these two major types of law. Political law contained systems where political relations can override law. Today, this type of law has narrowed to the socialist type of legal systems.^47^ Traditional law included legal systems in which law and religion are not separated, which means that secularisation has not taken place, unlike in political law and professional law. In this third group we find Islamic law, Hindu law and Confucian legal tradition—the list of usual suspects, if you like. Christianity was omitted as it has, one can only deduce, become an invisible fabric of professional Western law, which considers itself as fully secular.

Even though Mattei’s classification is different from the typical 20^th^-century macro-construction in its relation to religion, it nevertheless follows the mainstream of comparative law because he underlined the importance of the separation of law from religion: ‘the legal process is largely secularised. In other words, the legitimacy of the law is neither of religious nor of political origin, but rather of a technical nature.’^48^ In fairness, it must be said that Mattei did not argue that Western law would be thoroughly secular, as he describes the legal process as ‘largely secularised’. By and large, though, his hegemonic patterns place religion in non-Western systems. In that sense, this classification is partially blind in terms of perceiving religion in the fabric of modern Western law. Secularisation in this context refers to the separation of a society’s legal rules from the influence of religious institutions and the norms that are recognised by those institutions.^49^ In short, the link between law and religion belongs either to the past or to non-Western systems that are not (yet) secularised.

One of the problems with seeing secularism in this way is that it does not draw a distinction between legal systems which understand religious and worldly power as being fused and those which conceive law as enshrining religious values even though religious and worldly power are regarded as separate. In other words, the old school and its variants lack nuance in their ways of addressing the religious dimension in comparative study of law.

### B. Towards New Views?

An important exception in relation to other macro-constructions is Glenn’s volume on legal traditions of the world.^50^ Arguably, this tome has replaced Zweigert and Kötz’s work on major macro-constructs as the key work on the subject. Following mainstream scholarship, Glenn distinguished seven legal traditions, including chthonic (indigenous in a very broad sense), Talmudic, civil law, Islamic, common law, Hindu and Confucian. On the surface, this classification of traditions seems quite conventional as it puts different religious traditions into their own compartments. However, unlike others, Glenn recognises the role of religion in civil law and common law as well. In particular, the role of Christianity is stressed for the civil law tradition as human beings are created in the image of God, which means that the powers of man are reflections of the powers of God because humans ‘may exercise dominion over things, as does God over the world’.^51^ Instead of simply playing down the religious connection, Glenn makes it visible, albeit, first and foremost, in a historical context.

According to Glenn, the impact of the Judaeo-Christian tradition on civil law was enormous. For some reason, Glenn did not consider pre-Christian influences that may still run through civil law as he chose to underline the role of the Judaeo-Christian heritage. The most visible consequence is the centrality of the person as celebrated and cemented in civil law codifications: ‘There had to be rights, and there had to be codes to ensure their respect.’^52^ Because common law developed differently and separately from learned academic law and canon law, its relation to religion is different. Even though Glenn notes that English common law lawyers were likely quite religious people in the past, their law-related Christianity was not practised in the Crown Courts as it was limited to the ecclesiastical courts.^53^ But at the same time, Glenn notes how Christianity even today has a foothold in English common law, as can be seen in cases that concern blasphemy. He argues that common law to an extent protects Christianity, whereas other religions do not enjoy the same level of legal protection.^54^ Without delving deeper into this issue, it seems likely that this applies to most Western systems as their inferred relation to Christianity runs deep.^55^

In a nutshell, Glenn acknowledged common law’s connection to Christianity. This is not the same as to argue that common law has a religious foundation, even though in the 19th century US judges regularly explained that Christianity was part of the common law.^56^ It can even be argued that the Christian faith is considered as a primary source of US law. Furthermore, one can argue more broadly that most of the principles in the Ten Commandments are essential to the Western legal tradition. So, prohibitions on murder, theft and perjury are easily found in all legal systems, and ideas of respect for one’s parents and disapproval of adultery are implicit or, indeed, sometimes explicit elements of many legal systems.^57^ It goes without saying that the same principles are not alien to non-Western laws, either.

Importantly, Glenn did not argue that civil law and common law are religious legal traditions or that they would contain unconcealed religious influences or features to a significant degree. Even though Glenn’s approach seeks to be sensitive in relation to Western law and Christianity, he seems to relegate the role of religion to law’s past. Undoubtedly, religion was important for Western law’s path of development into what it is today. Indeed, Glenn made it clear that Western law is certainly not free from the ingrained impact of Christianity, even though today it is not customary to mention this in the context of macro-comparative law. Religious law is considered as the law of ‘the Other’, not of modern Western societies. However, attaching religion only to the laws of others implicitly suggests a non-neutral attitude. Using religion as a marker that tells Western law apart from systems that are anchored visibly to faith has a consequence that is crystallised by Monateri: ‘Religion becomes part of an orientalist strategy of exoticisation.’^58^ As a result, systems that are regarded as religious are implicitly painted as ‘exotic’.

Alongside Glenn’s, Menski’s thick volume differs from mainstream comparative law as it carefully avoids Eurocentrism and epistemic domination by Western law.^59^ Instead of discussing civil law and common law, Menski addresses Hindu law, Islamic law, African laws and Chinese law. His volume is based on ‘a methodological approach that integrates the social and ethical elements of law into a necessarily pluralist legal analysis to understand the pervasive role of law in its various social contexts’.^60^ In practice, this means that the relationship between law and religion is also taken into account when discussing each legal culture. Interestingly, though, Menski’s approach also connects religion with only non-Western systems as he leaves Western law out of the analysis altogether. This, in turn, may inadvertently strengthen the perception that Western law is secular and has no relevant connections with Christianity. This leads, bafflingly, to an overall impression that religion is relevant in non-Western systems but not in Western systems, although this is quite likely not what Menski wanted indirectly to convey.^61^

The above discussion leaves us with a question: what is the role of religion in macro-comparative law? All in all, as Michaels puts it, ‘religious law in comparative law is always more of an ornament than a real object of analysis’.^62^ It seems almost obvious that this is because macro-comparative law has focused on contrasts between civil law and common law ‘as it was apparently assumed that religious beliefs embodied in both types of legal systems were fundamentally the same, namely Christian’.^63^ This innate epistemic assumption leads to a scholarly imbalance that is particularly problematic for the comparative study of law, which seeks to strike a balance between the systems compared. Studies abound on Jewish law and on Islamic law, but there are very few on Christian law, even if compared with Buddhist law or Hindu law. What is more, in much of the study of Christian law the focus has been on Roman Catholic canon law.^64^ Epistemically, this may be expressed by paraphrasing the biblical quote from the beginning of this article: why do we see religion in the laws of others but fail to do so in terms of our own laws? For an Eastern viewer, however, Christianity is the key to understanding the core of Western law.^65^ In the eyes of a non-Western comparatist, the inherent influence that dominant Christian churches and groups exert on law is more visible and the presumed neutrality of state laws from religious values and norms seems blurred.^66^ This begs the question: has comparative law’s assumption of secularity rendered us blind to the religious roots and dimensions of Western laws? Is it not so that Western law has left religion in the past, where it rightly belongs?

## 3. Western Law and Christianity—a Thing of the Past?

For the most part, comparatists tend to separate common law and civil law. But if we focus on the common features of these two legal families, it is possible to speak of Western law without telling these families apart. Significant commonalities are based on human rights, notions of the rule of law, statutory law, legal professions and the separation between law/the state and religion. Accordingly, it is perhaps ‘justified to speak of a comprehensive Western legal tradition, being different from the laws of religious and tribal communities and dysfunctional states’.^67^ As I have already stated, the epistemic tendency to disregard the impact of Christianity on Western law most likely reflects a more general attitude among comparative law scholars. In essence, comparatists seem instinctively to keep a distance from theology as they feel closer to the doctrinal study of law or even social sciences. This is understandable if we take into account the difficulties that the comparative study of law faces when it seeks to become an interdisciplinary field.^68^ Theology may feel too close to a past that secular comparative law hopes to have left behind a long time ago. To be sure, theology is not even mentioned when the neighbouring disciplines of comparative law are discussed.^69^ The underlying narrative of Western comparative law seems to suggest that ‘we are not religious, at least not any longer’. As a result, Western comparatists do not see their own state laws as falling into the religion-influenced category where they place Islamic, Jewish, Hindu and canon law.^70^

There are, nonetheless, examples of how Christianity has shaped substantive areas of law, even though most of this body of scholarship is not manifestly comparative in terms of its nature.^71^ That said, recently there have been attempts to cross the borders of legal systems and discuss how Christianity has also shaped our understanding of so-called global law.^72^ Taking into account the transnational character of Christianity, the border-crossing approach seems a potentially fruitful area for comparative research. It is no surprise, then, that some of the literature draws parallels between secular and religious laws, offering comparative points of view across religions.^73^

As discussed above, the chosen epistemic point of view plays a role here. Hadrianto has argued that comparative study of law may suffer because non-religious scholars consider them as outside observers. He goes further and claims that comparatists are reluctant to consider, and perhaps even hostile towards, the religious viewpoint.^74^ From a historical perspective, the idea of secular Western law is curious because one can argue that, for centuries, law and religion have formed part of the intellectual pedigree of the Western Christian tradition.^75^ Kischel rightly notes that ‘many aspects of diverse state legal orders, including common law, have been derived from canon law’.^76^ Notwithstanding, it is not sufficient for macro-comparative law to dismiss the significance of religion as a thing of the past.^77^ Arguing that Christianity was important for the development of Western law is certainly not wrong, but it does exclude discussion on what the situation is today. A reasonable assumption is that religion-related subtleties disappear when law is exhibited under the banner of secular professional law.

An illustration of comparative law and religion is presented by Siems, who addresses family law and consumer credit as areas where Christian values still seem to play a role in many legal systems.^78^ Importantly, Siems is not arguing that family law and consumer credit law reflect only Christian values because usury prohibitions may be based on the practicalities of societies that stress the importance of reciprocity elsewhere.^79^ Darian-Smith, in turn, has questioned the underlying Western assumption that modern law is a purely objective, rational and secular phenomenon. She makes a strong point that, in fact, in Anglo-American law, common law’s rule of law principle is historically grounded and connected to the particularities of Christian morality and capitalism. In particular, she highlights how Western notions of individualism stemmed from the Reformation in the 16th century, later developing in the following centuries under the influence of the Enlightenment.^80^ Darian-Smith’s viewpoint is historical, but beneath it lies a far-reaching attempt to show the influence of Christianity on Western law by stressing ‘the sacred, irrational and ideological elements embodied in law’.^81^ Of course, the varieties of Christianity that have been debated and integrated into US law offer only a limited view on the role of Christianity in Western law. Yet, even more general implications are possible if the door between law and religion is opened.

Beyond the foundations of Anglo-American law, Western family law and consumer credit, more obvious signs can be found of the significance of Christianity for Western law and legal thinking. One such area is constitutional law. For instance, the Preamble of Poland’s constitution of 1997 speaks of belief ‘in God as the source of truth, justice, good and beauty’ and ‘responsibility before God’.^82^ The Russian constitution was amended in 2020 so that Article 67 now speaks of ‘the Russian Federation, united by a millennium of history, preserving the memory of the ancestors who conveyed to us ideals and belief in God’.^83^ Amended Article 67 has a broad theological background as it refers not only to the history of the Russian state and Christianity in Russia, but also to the historiosophical fiction of Moscow as the third Rome. This idea holds, essentially, that Russia is seen as the sole country of legitimate Christianity and Russians as a God-fearing people.^84^ However, it can be argued that the Russian notion of law and Christianity does not coincide with that of Western notions.^85^ Nevertheless, Russian law’s religious nature cannot be denied. At the same time, these accounts fail to address divergences between Christian principles and law. Such divergences may seem to run counter to the core thesis of this article. However, as the focus is on comparative law scholarship, these disparities are not discussed here.^86^

It is possible that Hadrianto has a point as to the reluctance and hostility of comparatists. However, as I see it, the lack of a religious view as such is not the core problem with macro-comparative law and religion. Rather, the issue concerns epistemic bias, because Western comparatists chart religious influence in other systems but fail to do so when it comes to their own legal tradition. This is not hostility or even reluctance, but has to do with the fact that Western societies are seemingly so secularised that the intimate connection between Christianity and law has become not so much less palpable as invisible. Invisibility has consequences for the epistemic assumptions that are implicitly embedded in the comparatist’s *modus operandi*. Somewhere along the road, Western comparatists lost their ability to recount the religious dimension in their own contemporary law.^87^

The question, then, is what should comparatists do differently? Instead of needing to become more religious or applying a more theological viewpoint, comparatists should be able to distinguish between two things: *recognisable religiousness*, as in Islamic, Hindu and Jewish law, and *unrecognisable religiousness*, which lies hidden beneath the surface level of Western law. Importantly, it should be noted that even though recognisable on the basis of its supernatural elements, religious law may also possess a legal character.^88^ Consequently, telling these dimensions apart may be more difficult than is normally assumed and there are differences between the various fields of law.

To be able to detect unrecognisable religiousness requires the researcher to maintain the epistemic position of an outside observer, while at the same time being able to recognise the impact of Christian theology on law. The comparatist who is to learn from theology without transforming into a quasi-theologist must be active within a secular and non-religious framework that keeps the comparatist’s beliefs away from comparison but at the same time does not fail to chart the effect of religion on their own epistemic home base in the shape of Western law. This is important because comparative law has more or less ‘lost its consciously Christian colouration’.^89^ This does not mean that colouration would not exist regardless of whether or not comparatists themselves realise that Christianity is an embedded factor. For all these reasons, nuances are important when discussing Christianity and law, as differences exist within Western law even though the Western religious tradition is by and large similar to non-Western traditions. For instance, as Siems points out, in the United States, Christian values in terms of law can be seen that are based on the Old Testament idea of retribution, whereas this is not the case in Germany, as these two Western criminal law systems seem to hold different visions of humanity’s moral nature.^90^

The epistemic move of comparative law paving the way towards the disappearance of Christianity is probably quasi-theological and has to do with the fact that—during its development towards today’s forms—Christianity confined ritual practices to within the church. This, in turn, made it only natural for Western comparatists to envisage certain compartments of society as ‘religious’ while conceiving other parts as ‘secular’.^91^ This probably explains why Western comparatists easily see religion in systems that incorporate significant parts of their law from Islamic, Hindu, Jewish or Buddhist legal traditions. As a result, Western legal rules and principles that developed in an intimate relationship with Christianity are assumed to be ‘universal’ or ‘general’. By the same token, civil law, common law and systems combining these two are conceived as non-religious systems because they are easy to place into a secular compartment. For example, when Islamic legal culture is described, comparatists typically draw a line between ‘moral’ and ‘legal’. Consequently, when speaking of Islamic legal culture, Western comparatists may point out that ‘In this legal culture moral principles have more weight than rational, systematic legal constructions’.^92^ The underlying theoretical framework seems to be that of *evolution of law*; Western law is deemed to have developed further along its path from the shackles of religion towards secular law.^93^ The epistemic problem is that religious law becomes a distinct type of law that is *produced* by macro-comparative law rather than studied as a phenomenon which also concerns legal systems that are conceived as secular.

There is more to it, however, because placing Western law into a non-religious compartment omits acknowledging that the very distinction between secular and religious forms of law comes from Christian religious notions as it echoes the distinction between church and state.^94^ What is more, Christian doctrines evidently now form part of Western secular law. An illustration of this is the right against self-incrimination. Medieval law of the Roman church recognised the maxim *nemo tenetur prodere seipsum*, which essentially provided that a Christian’s duty of confession did not go as far as instituting or inviting criminal proceedings against themselves. One could confess a sin to a priest, but one was not obliged to confess a punishable offence to a court of law or prosecutor.^95^ Today, the right against self-incrimination is a celebrated part of many legal systems. An important example of this criminal and procedural law principle is the right to a fair trial, as is provided by the European Convention on Human Rights, Article 6.^96^ This example illustrates how Christian doctrines have transformed into ‘purely’ legal doctrines.^97^ But we can go further. The impact of Christian canon law goes well beyond singular examples, and it can be argued that ‘Canonists created most of the high medieval innovations, many of which still characterise modern criminal law’.^98^ These considerations open up the question how should the comparatist tackle the problem of seeing beyond the secular facade?

## 4. Seeing Below the Secular Surface

Even though one might assign less weight to the past, as is suggested here, history still provides useful insights. Before the development of secularism started in continental Europe, religious texts were important not only for theologists, but also for lawyers, as these texts contained behavioural norms.^99^ Regardless, as the separation of church and state was already underway, legal scholarship built on the remains of Roman law, which was conceived as something ‘legal’ but not ‘religious’.^100^ Later, the Protestant reformation further developed the idea of the separation of church and state, thus foreshadowing the societal separation of the religious from the secular.^101^ This is different from Islamic law, where legal theories and the idea of the state developed differently, leading to a situation in which religious authorities may even today enjoy special status beyond the reach of official state law.^102^ While one might feel tempted to argue that this exception concerns only non-Western systems, nevertheless some Western seemingly secularised states have a close relation with their Protestant, Catholic and Orthodox churches.^103^ As the present link between law and Christianity is not apparent, some scholars see it as having receded whereas others see Christian principles as still suffusing law.^104^

In principle, it is easy to see what separates Western law from non-Western law: Judaeo-Christian moral principles, individualism leading to rights-based thinking and the separation of law from religion.^105^ Roman law should, of course, be added to this list. The point that bears repeating here is that conceiving these things as purely legal seems problematic when comparatists at the same time consider religion as a virtually overwhelming factor in non-Western law. One feature of Christianity is that it pronounces a special view of human beings and their societies, which, in turn, involves unavoidable ramifications not only for Christians, but also for those who live in cultures and societies that are shaped by the Christian faith and Christian culture.^106^ The effect of colonisation on law around the globe was (and still is) enormous, even if today’s comparative law scholarship struggles to elude its repercussions.^107^ To that end, decolonial comparative law also warrants a critique of secularism as a hegemonic and universalising paradigm of comparative law scholarship.^108^ For example, the Western notion of marriage as in principle a life-long institution is thoroughly Christian and the very reason why fixed-term marriage feels alien to Western lawyers.^109^ With that in mind, it should not be difficult to grasp why Christianity has an everlasting and continuing impact on Western law. For example, such a foundational contract law principle as *pacta sunt servanda*, according to which ‘agreements must be kept’, has deep roots in religion and morals built on a religious base; the underlying idea is that of the *sanctity* of contract.^110^

In a more nuanced historical view, however, the sanctity of contract seems to be derived from the general Christian idea according to which breaking a promise is in itself a sin.^111^ In any case, the interaction between the legal and the religious is clear. Notwithstanding, the Christian doctrine supporting the principle according to which ‘promises should be kept’ has its deep roots in the sacred and philosophical ideas of pre-Christian antiquity, extending from ancient Greece to Mesopotamia.^112^ The interaction between Christian and pre-Christian sacred values within Western legal systems is a relevant area to be explored, but it is beyond the scope of this discussion as the focus is on comparative law’s feeble interest in Christianity on the level of macro-comparative law. The caveat here is, of course, that Christianity is conceived broadly so that it may also include elements that were imported from the previous cultures.

The challenge for the comparatist is tricky because it is not an easy task to see clearly how to distinguish between religion as *an element* of law and religion as *an influencing factor* of law.^113^ The same difficulty is to be found not only in Western law, as the same distinction is also blurred, for example, in Islamic law and its relation to the state.^114^ This blurred distinction has been reflected in comparative law’s reluctance to study family law. For instance, Zweigert and Kötz recommended avoiding family law because it was, according to them, ‘heavily impressed by moral views’.^115^ Along similar lines, family law was deemed a problematic area for comparative study of law because it was associated with non-legal factors, including religion.^116^

In view of the above narrative, what is suggested here is not to overemphasise Christianity in contemporary Western law, but rather to attempt to recognise subtleties that involve religion but are not purely religious.^117^ It would certainly not make much sense to label Western law as purely Christian. This is about recognising religion as an influential factor in the law, not about subjugating comparative law to theology or a religious framework. Clearly, Western law is heavily secularised, but it would be useful to see the nuances and differences between the various fields of law. For instance, there is very little doubt that contemporary Western family law or criminal law contain more Christian theological elements than company law or tax law.

Similar considerations arise in the case of abortion laws, showing how controversies concerning abortion are not divided between religious and secular authority as grey zones exist where socio-political circumstances are mixed with religious dimensions.^118^ Taking Christian elements into account does not mean classifying Western law as religious; rather, it means taking into account religious dimensions when relevant to do so. One matter I want to emphasise is that this also entails the need to tone down the distinction between secular and religious systems, as no legal system is either thoroughly secular or entirely religious. Secular and religious elements interact with varying degrees of centrality. As Doe puts it, ‘religious law exists as a legal fact in society today—in both guises, classical and modern’.^119^ This is not to suggest that religion, or a revelation that is supposed to be the truth on which a religion is based, would be used directly as a source of law in Western law, as religious and legal dimensions are intermingled.^120^ Along similar lines, Michaels has proposed a post-secular comparative law that would need to identify and handle religious law as law simply ‘because it functions as law’.^121^

There is one further point to be made here: influences also move from secular Western law to modern Christian law as interpreted through various Christian traditions (such as Catholic, Orthodox, Anglican, Lutheran, Methodist, Reformed, Presbyterian and Baptist), demonstrating the existence of the nascent field of comparative church law.^122^ The comparative study of laws across the various Christian traditions is doubtless an important contact point between comparative law and Christianity, even though it does not have an established place in modern comparative law scholarship.^123^

## 5. Discussion

Finding a balance between the religious (spiritual, mystical, sacred) and the rational (secular, legal) within the legal-cultural sphere of Western law has been understood as a *fait accompli*, in that the rational has emerged as the winner whereas the religious has been pushed into the shadows of modern law, never to be seen again. That is to say that Western law’s evolution is seen as more developed than non-Western law. Comparative law scholars involved in macro-comparative law have inherently accepted this as a fact that has not caused them to consider the accompanying epistemic limitations with a critical eye. From the surface, this seems to make perfect sense, as the relation between, for example, Islam or Hinduism to law is visible because it is anchored to positive law, as can be seen in, for instance, the Constitution of Iran^124^ or the Indian Hindu Marriage Act.^125^ Christianity, for its part, lurks beneath the surface.

Nevertheless, in the world of law, looks can be deceiving. This, if anything, is the key takeaway from the diverse and voluminous comparative law scholarship of recent decades. Consequently, if the Western comparatist were to look at their own law with a more sensitive eye, the assumption of *fait accompli* might need to be reconsidered because the relation between the religious and the legal is subtle, even opaque. As Witte points out: ‘Law and religion are distinct spheres and sciences of human life, but they exist in dialectical interaction, constantly crossing-over and cross-fertilising each other.’^126^ To that extent, it feels safe to argue that it is this dialectical interaction that needs to be more openly recognised than is the case today. My claim here is that macro-comparative law would benefit from self-critical evaluation, allowing room to recount the influence of religion on Western law.

If the comparatist is not raised on the implicit Christian assumptions that shape our underlying conceptions and epistemology, then it becomes easier to grasp the impact of Christianity on law even though Western societies appear entirely secularised.^127^ However, the fact that Christian theological references are now omitted from legal texts and scholarly discourse does not mean that people familiar with theology would not be able to recognise the religious dimension. The problem for comparative law is that religious dimensions can mingle with political dimensions in terms of views on law.^128^ This all seems not legally relevant, or at least something that no longer plays a role in Western law. As a result, the need arises to reassess macro-comparative law and the way in which it depicts Western law as the *opposite* of religious law. Needless to say, macro-comparative law is based on generalisations, but some subtlety would be useful. Perhaps macro-comparison has reached the end of the road and the future is for more nuanced classifications based on fields of law and thematic areas rather than entire legal systems. In a similar vein, it has been argued that legal families and similar macro-concepts are problematic conceptualisations, and they do leave plenty of room for criticism, though that cannot be discussed further here.^129^

Despite recognising the importance of religion for Western legal culture, one needs to tread carefully in order to avoid an overly simplistic takeaway. At the very least, it is useful to seek a nuanced approach to law and religion so that the legal scholar is not blind to their own law’s religious dimensions and merely detects a religious flavour only in the laws of others. Essentially, comparative law does not need to transform into comparative study of religious law; instead, comparative law needs to shape its epistemic scope to chart the religious dimensions of Wester law. There is something unbalanced in the manner in which comparatists are able to unmask religion as an element in foreign legal systems but fail to do the same when it comes to their own law. The first step here would be to admit that the relationship between law and religion in Western law is not an obsolescent—if not obsolete—historical relic but in some places underpins the living law of today. Metaphorically, this would mean seeing the plank in our own eye. Ultimately, it may be the case that all legal systems are religious to an extent, so that the real challenge is to recognise this dimension as a contextually relevant factor in the comparatist’s own law.

The observations I have made in this article should give cause for a more nuanced, reflexive and contextual approach to macro-comparative law. Paradoxically, perhaps, becoming more nuanced, reflexive and contextual may mean that no room remains for meaningful macro-comparative law beyond the classroom. The broad implication is that macro-concepts are not overly scientific or exact constructions, but they do provide law students with an initial formative framework for organising their perception of law in general.^130^ It is for this reason that Western comparatists should recount the religious elements in their own law and not only in the laws of the exoticised ‘Other’. Perhaps law has become a religion per se in the West, that is, replacing much of the space that Christianity used to fill, which in turn may explain why overtly religious law looks so peculiar to the comparatist.^131^ In any case, when our human environment has transformed into an intercultural space where diverse visions of the world live together, it is time to reconsider how we construct and present the global world of law and how we see religion in the overall picture.

## Footnotes

1 There are different versions using different words for ‘speck’ (kάρφος) and ‘plank’ (δοκός)—for instance, ‘mote’ or ‘chaff’ and ‘log’ or ‘beam’. See eg GB King, ‘The Mote and the Beam’ (1924) 17 Harvard Theological Review 393; JDM Derrett, ‘Christ and Reproof (Matthew 7.1–5/Luke 6.37–42)’ (1988) 34 New Testament Studies 271. What is clear, in any case, is that a beam/log/plank is a large piece of wood whereas speck/mote/chaff refers to a tiny piece of wood. Crucially, speck and plank are of the same substance.

2 See eg Å Malmström, ‘The System of Legal Systems: Notes on a Problem of Classification in Comparative Law’ (1969) 13 Scandinavian Studies in Law 127; M Pargendler, ‘The Rise and Decline of Legal Families’ (2012) 60 Am J Comp L 1043.

3 See J Vanderlinden, *Comparer les droits* (Diegem, Kluwer, 1995) 311–37; J Husa, ‘Classification of Legal Families Today: Is It Time for a Memorial Hymn?’ (2004) 56 Revue internationale de droit comparé 11; M Siems, ‘Varieties of Legal Systems: Towards a New Global Taxonomy’ (2016) 12 Journal of Institutional Economics 579; U Kischel, *Comparative Law* (A Hammel tr, OUP 2019) 201–24.

4 R Michaels, ‘Religiöse Rechte und postsäkulare Rechtsvergleichung’ in R Zimmermann (ed), *Zukunftsperspektiven der Rechtsvergleichung* (Mohr Siebeck 2016) 39, 43–55.

5 See eg J Smits, *Elgar Encyclopedia of Comparative Law* (2nd edn, Edward Elgar 2012), with no entries on religious law, whereas M Bussani and U Mattei, *The Cambridge Companion to Comparative Law* (CUP 2012) has chapters on Jewish and Islamic legal traditions.

6 The problem is the same with all religions, although it may be the case that frameworks constructed for law and religion work with the Abrahamic monotheistic religions but are problematic for other kinds of religion, such as Buddhism and Hinduism. See A Welikala, ‘Theorising Constitutionalism in Buddhist-Dominant Asian Polities’ in T Ginsburg and T Schonthal (eds), *Buddhism and Comparative Constitutional Law* (CUP 2023) 57, 62.

7 S Hendrianto, ‘Comparative Law and Religion: Three-Dimensional (3D) Approach’ (2017) 7 Law and Method 1.

8 For characteristics of mixed systems, see VV Palmer, ‘Introduction to Mixed Jurisdictions’ in VV Palmer (ed), *Mixed Jurisdictions Worldwide: The Third Legal Family* (2nd edn, CUP 2012) 3, 7–11.

9 HJ Berman, ‘Comparative Law and Religion’ in M Reimann and R Zimmermann (eds), *The Oxford Handbook of Comparative Law* (OUP 2006) 739, 741.

10 Blindness may be self-chosen, though not always. For instance, one can say that there are ‘non-Western conceptions of law, such as Muslim, Hindu, Chinese, and Japanese conceptions of law. These are based on religious beliefs or customary law. It is not proposed to discuss these laws here’, P de Cruz, *Comparative Law in a Changing World* (3rd edn, Routledge-Cavendish 2008) 209.

11 JQ Whitman, ‘Comparative Law and Religion’ in M Reimann and R Zimmermann (eds), *The Oxford Handbook of Comparative Law* (2nd edn, OUP 2019) 734, 735.

12 It has been argued that legal scholars should pay more attention to religion, see eg R Sandberg, *Religion, Law and Society* (CUP 2014).

13 J Husa, *Introduction to Comparative Law* (2nd edn, Hart Publishing 2023) 106–8. See also M Siems, *Comparative Law* (3rd edn, CUP 2022) 14.

14 See M Hill, ‘Legal Theology’ (2017) 32 Journal of Law and Religion 59, 60 (‘Legal theology is the investigation of theological ideas as used by legislators, administrators, and judges in their decision making, and in the legal texts they produce’).

15 Hendrianto (n 7) has suggested coupling comparative law with theological insight, thus inviting comparatists to engage in theological studies. This article discusses religion and especially Christianity, but the point here is epistemic as the focus is on macro-comparative law.

16 Of course, there are several potential connection points between law as a discipline and theology, including ethics, rhetoric and interpretative methods. For a more detailed discussion, see M Welker, ‘What Could Christian Theology Offer to the Disciplines of the Law?’ (2017) 32 Journal of Law and Religion 46 (‘Christian theology can unfold the deep foundations of a cousinly relationship between law and religion, theological and legal thinking’, 52).

17 RB Hays, ‘Whose World’ Which Law?’ (2017) 32 Journal of Law and Religion 17.

18 cf Hendrianto (n 7) 5–7. See also B Clark, ‘A Comparative Method for the Study of Law and Religion: Is This a Defensible Methodology?’ in R Sandberg and others (eds), *Research Handbook on Interdisciplinary Approaches to Law and Religion* (Edward Elgar 2019) 341, 341–2.

19 See eg S Fredman, *Comparative Human Rights Law* (OUP 2018) 401–56.

20 See eg A Huxley (ed), *Religion, Law and Tradition: Comparative Studies in Religious Law* (Routledge 2022) ch 3, which is devoted to Roman Catholic canon law. See also Silvio Ferrari, *Lo spirito dei diritti religiosi: Ebraismo, cristianismo e islam a confront* (Il Mulino 2002), in which Christianity and law is limited to the Roman Catholic canon.

21 Kischel (n 3) 866.

22 See J Esposito and others, *World Religions Today* (7th edn, OUP 2021).

23 Siems (n 13) 82–3.

24 Husa, *Introduction to Comparative Law* (n 13) 163 points out that in 20th-century mainstream comparative law ‘the study of the relation between religion and law was not held in very high esteem. Even in extensive works the practice was usually to simply dismiss the significance of religion by referring to it in only a very small number of pages.’ See also M Bogdan, *Komparativ rättskunskap* (Norsteds 2003) 68 (religion is one of the factors that are used when differences and similarities are explained).

25 According to Fredman (n 19) 347, ‘The comparative perspective provides a vista into other nations’ ways of dealing with clashes and reconciliation between law and religion. Thus, an international perspective may also reveal commonly shared values and norms and assist nations in dealing with these issues.’

26 cf Michaels, ‘Religiöse Rechte’ (n 4) 45.

27 cf Berman (n 9) 745–6.

28 Kischel (n 3) 789. Other forms discussed as religious traditions by Kischel are Jewish law and canon law (852–67), with an explanation that ‘their practical importance is limited, which explains the minor role they play in comparative law studies’. Kischel also addresses Hindu law in the context of Indian law (752–73).

29 L Salaymeh and S Lavi, ‘Religion is Secularised Tradition: Jewish and Muslim Circumcisions in Germany’ (2021) 41 OJLS 431, 436.

30 See eg S Koch and J Øyrehagen Sunde (eds), *Comparing Legal Cultures* (2nd edn, Fagbokforlaget 2020). Legal cultures addressed are Norwegian, Finnish, Estonian, German, Polish, English & Welsh, Scottish, Belgian, French, Austrian, Italian, Romanian, American, Australian and Chinese.

31 See N Doe, *Comparative Religious Law: Judaism, Christianity, Islam* (CUP 2018). The book addresses laws and other regulatory instruments of Jewish, Christian and Muslim organisations in the UK, with a particular focus on England and Wales.

32 M Reimann, ‘The Progress and Failure of Comparative Law in the Second Half of the Twentieth Century’ (2002) 50 Am J Comp L 671, 679.

33 Berman (n 9) 739.

34 K Zweigert and H Kötz, *An Introduction to Comparative Law* (3rd edn, T Weir tr, OUP 1998).

35 ibid 303–19.

36 ibid 74–302.

37 ibid 113–14.

38 Whitman (n 11) 735.

39 eg Wigmore distinguished 16 groups of law: Egyptian, Mesopotamian, Hebrew, Chinese, Hindu, Greek, Roman, Maritime, Japanese, Mohammedan, Keltic, Germanic, Slavic, Ecclesiastical, Romanesque and Anglican. See JH Wigmore, *A Panorama of the World’ s Legal Systems*, vols 1–3 (West Publishing 1928). The significance of Catholic and Orthodox canon law and Protestant law is discussed by P Arminjon, B Nolde and M Wolff, *Traité de droit comparé*, vol 1 (LGDJ 1950) 63–79. Their discussion concerns the civil law legal family.

40 See eg H McLeod, *The Religious Crisis of the 1960s* (OUP 2007) 215–39 (discussing the decline of Christendom and the move towards a more pluralistic society).

41 R David, *Les grands systèmes de droit contemporains* (Dalloz 1964). Numerous translations and later editions have appeared since, despite the fact that David died in 1990. His name has been preserved as an author alongside M Goré and C Jauffret-Spinosi; the 12th edn came out in 2016.

42 G Cuniberti, *Grands systèmes de droit contemporains* (4th edn, LGDJ 2019).

43 ibid 287–334.

44 ibid 368–75.

45 ibid 394–5.

46 U Mattei, ‘Three Patterns of Law: Taxonomy and Change in the World’s Legal Systems’ (1997) 45 Am J Comp L 5.

47 Socialist legal systems are, in practice, combinations of socialist law and other kinds of elements. For example, in the People’s Republic of China, the socialist element is seen in the Party leadership implemented through governance based on the narrow idea of rule of law (‘accordance with the law’). See D Lin and S Trevaskes, ‘Creating a Virtuous Leviathan: The Party, Law, and Socialist Core Values’ (2019) 6 Asian Journal of Law & Society 41.

48 ibid 23.

49 cf S Lettmaier, ‘Marriage Law and the Reformation’ (2017) 35 Law & History Review 461, 464.

50 HP Glenn, *Legal Traditions of the World* (4th edn, OUP 2014).

51 ibid 150. However, ‘Secular law’s demand for positive law, in the form of a divinely ordained legal code, is far from neutral’, Salaymeh and Lavi (n 29) 449.

52 Glenn (n 50) 152. However, the division of jurisdiction between the king’s courts and the church courts was not clear and differs from our modern notions. See PD Jason, ‘The Courts Christian in Medieval England’ (2017) 37 The Catholic Lawyer 339, 351–4 (discussing the pre-Reformation era).

53 Glenn (n 50) 254.

54 ibid 268.

55 One example is how European courts may treat religious symbols of Christianity and Islam differently, raising doubts about double standards. See C Joppke, ‘Double Standards? Veils and Crucifixes in the European Legal Order’ (2013) European Journal of Sociology 97.

56 For a more detailed discussion, see S Banner, ‘When Christianity Was Part of the Common Law’ (1998) 16 Law & History Review 27.

57 SK Green, ‘The Fount of Everything Just and Right? The Ten Commandments as a Source of American Law’ (2000) 14 Journal of Law and Religion 525. See also Berman (n 9) 747.

58 PG Monateri, *Advanced Introduction to Comparative Legal Methods* (Edward Elgar 2021) 30.

59 W Menski, *Comparative Law in a Global Context: The Legal Systems of Asia and Africa* (2nd edn, CUP 2006).

60 ibid 7.

61 Essentially, ‘Religious law is often understood to be fundamentally different because of its religious nature’, R Michaels, ‘On the Comparability of Religious Law’ (2022) Ancilla Iuris 19, 38.

62 Michaels, ‘Religiöse Rechte’ (n 4) 41 (‘Im Großen und Ganzen sind religiöse Rechte für die Rechtsvergleichung immer noch mehr Ornament denn echtes Objekt’).

63 Berman (n 9) 739–40.

64 cf Doe (n 31) 6–7.

65 B Grossfeld, *The Strength and Weakness of Comparative Law* (Clarendon Press 1990) 108.

66 See eg A Aftab, ‘Religious Accommodation in the Secular State: The Sharia Debates in Australia, Canada, and the United Kingdom’ (2019) 79 SLPS 85.

67 Siems (n 13) 77.

68 For a more detailed discussion on interdisciplinarity in comparative law, see J Husa, *Interdisciplinary Comparative Law* (Edward Elgar 2022).

69 See eg M Reimann, ‘Comparative Law and Neighbouring Disciplines’ in M Bussani and U Mattei (eds), *The Cambridge Companion to Comparative Law* (CUP 2012) 13. See also Husa, *Interdisciplinary Comparative Law* (n 68), discussing history, linguistics, literature, economy, sociology and close fields of laws (public and international private law, global law) but leaving religion out.

70 Grossfeld (n 65) 107.

71 M Hill and others (eds), *Christianity and Criminal Law* (Routledge 2020) offers historical, theological, philosophical and law-related perspectives on Christianity and criminal law. F Longchamps de Bérier and R Domingo (eds), *Law and Christianity in Poland: The Legacy of the Great Jurists* (Routledge 2023) studies how Christianity played a part in shaping the evolution of law in Poland by focusing on selection of central historical figures in Polish law.

72 R Domingo and J Witte Jr (eds), *Christianity and Global Law* (Routledge 2020). This book asks border-crossing questions such as how to understand global law and whether global law can be based on Christian principles.

73 S Ferrari and R Bottoni (eds), *Routledge Handbook of Religious Laws* (Routledge 2019) addresses Christian, Jewish, Islamic and Hindu traditions.

74 Hadrianto (n 7) 6.

75 Whitman (n 11) 737.

76 Kischel (n 3) 861.

77 This is, by and large, the argument by Michaels, ‘Religiöse Rechte’ (n 4), as he suggests that we should give more weight to living religious law(s) and treat the relationship between secular and religious law essentially as a conflict of laws (*Rechtskollision*).

78 For more detailed analysis on markets and law, see DA Crane and S Gregg (eds), *Christianity and Market Regulation: An Introduction* (CUP 2021). The book discusses the relation between Christianity and such issues as bankruptcy, corporate law and antitrust law.

79 Siems (n 13) 181.

80 E Darian-Smith, *Religion, Race, Rights: Landmarks in the History of Modern Anglo-American Law* (Hart Publishing 2010).

81 ibid 2.

82 The Constitution of the Republic of Poland, 2 April 1997, as published in Dziennik Ustaw No 78, item 483.

83 The Constitution of Russia, as amended 1 July 2020.

84 J Sadowski, ‘Amendments of 2020 to the Russian Constitution as an Update to Its Symbolic and Identity Programme’ (2022) 35 International Journal for the Semiotics of Law 723, 729.

85 In short, contemporary liberal constitutionalism requires secularism, see M Rosenfeld, ‘Constitutionalism and Secularism: A Western Account’ in S Mancini (ed), *Constitutions and Religion* (Edward Elgar 2020) 21.

86 An obvious historical example is usury, see CF Taeusch, ‘The Concept of “Usury”: The History of an Idea’ (1942) 3 Journal of the History of Ideas 291; JL Bernstein, ‘Background of a Gray Area in Law: The Checkered Career of Usury’ (1965) 51 ABAJ 846.

87 Welker (n 16) 46 asks humorously: ‘Does the attempt of Christian theology to have an impact on disciplines of the law not provoke a single response, namely “Run for your lives!”’

88 cf Doe (n 31) 392.

89 Whitman (n 11) 737.

90 Siems (n 13) 155.

91 cf Whitman (n 11) 749.

92 M Van Hoecke and M Warrington, ‘Legal Cultures, Legal Paradigms and Legal Doctrine: Towards a New Model for Comparative Law’ (1998) 47 ICLQ 495, 505. Yet, Van Hoecke and Warrington are not blind to the significance of the epistemic point of view, as they also note that ‘Asian legal culture, when interpreted from an (overtly) Western point of view, can to a certain extent be represented as irrational, because of the important role of morals, or religion and of the Confucianist conception of the natural order of things’, ibid 505–6.

93 Husa, *Introduction to Comparative Law* (n 13) 253 notes that ‘In the early 1900s, scholars believed by and large that the development of law went through a series of stages, ie from a non-Western uncivilised form towards Western civilised forms’. This idea has its roots in late 19th- and early 20th-century sociological theory (eg Durkheim, Weber). In the context of religion, see eg R Boudon, ‘Max Weber on the Rationality of the Religious’ (2001) 51 L’Année sociologique 9.

94 cf Berman (n 9) 740.

95 See eg JH Langbein, ‘The Historical Origins of the Privilege Against Self-Incrimination at Common Law’ (1994) 92 Mich L Rev 1047, 1072.

96 See eg N Vogiatzis, ‘Interpreting the Right to Interpretation under Article 6(3)(e) ECHR: A Cautious Evolution in the Jurisprudence of the European Court of Human Rights?’ (2022) 22(1) Human Rights Law Review.

97 Of course, Christianity’s impact on criminal law goes well beyond this. It has been pointed out that the very notion of individual guilt comes from Christian theology. For a more detailed discussion, see H Pihlajamäki, ‘Christianity and the Liberal Enlightenment Reforms of Criminal Law’ in Hill and others (n 71) 80 (when criminal law was modernised Christianity stopped serving as a background ideology of criminal law, ie criminal law was not an instrument to channel God’s retribution on sinners. Regardless, the idea of individual responsibility and its different degrees—invented in canon law—survived the impact of the Enlightenment).

98 H Pihlajamäki and M Korpiola, ‘Medieval Canon Law: The Origins of Modern Criminal Law’ in M Dubber and T Hörnle (eds), *The Oxford Handbook of Criminal Law* (OUP 2014) 201, 203. Canonists worked hard to be able to explain and construct the Christian notion of ‘sin’ and its connection to criminal law.

99 See RC Van Caenegem, *Judges, Legislators and Professors* (CUP 1987) 110–11.

100 ibid 126. In time, however, the notions of Roman and Christian became synonymous, B Wauters and M de Benito, *The History of Law in Europe* (Edward Elgar 2017) 38–9.

101 Areas of law that transformed beyond church–state relations were religious freedom, marriage and family law, education and social welfare. Some areas of private law and criminal law were also affected by Protestant theology. See J Witte Jr, ‘Law and the Protestant Reformation’ in H Pihlajamäki, M Dubber and M Godfrey (eds), *The Oxford Handbook of European Legal History* (OUP 2018) 583.

102 A Black, H Esmaeili and N Hosen, *Modern Perspectives on Islamic Law* (Edward Elgar 2013) 5–6.

103 See eg R Sandberg and C Doe, ‘Church–State Relations in Europe: From Legal Models to an Interdisciplinary Approach’ (2008) 1 Journal of Religion in Europe 329.

104 See J Rivers, ‘Is English Law Still Christian?’ in N Spencer (ed), *Religion and Law* (Theos 2012) 143; J García Oliva and H Hall, *Religion, Law and the Constitution: Balancing Beliefs in Britain* (Routledge 2018).

105 cf V Zeno-Zencovich, *Comparative Legal Systems* (2nd edn, Roma TrE-Press, 2019) 99.

106 cf DA Crane and S Gregg, ‘Introduction’ in DA Crane and S Gregg (eds), *Christianity and Market Regulation: An Introduction* (CUP 2021) 1, 4–5.

107 L Salaymeh and R Michaels, ‘Decolonial Comparative Law: A Conceptual Beginning’ (2022) 86 Rabels Zeitschrift für ausländisches und internationales Privatrecht 166.

108 ibid 185.

109 cf J Smits, ‘On Lifelong and Fixed-Term Marriage’ in SP Donlan and J Mair (eds), *Comparative Law: Mixes, Movements, and Metaphors* (Routledge 2020) 87, 88–9.

110 For a more detailed analysis, see H Wehlberg, ‘Pacta Sunt Servanda’ (1959) 53 AJIL 775, 786 (‘the principle of sanctity of contracts is an essential condition of the life of any social community’).

111 HJ Berman, ‘The Religious Sources of General Contract Law: An Historical Perspective’ (1986) 4 Journal of Law and Religion 103, 109–10 (‘Our contract law started … from the theory that a promise created an obligation to God’, ibid 112).

112 For instance, the first systematic theory of contract law was a synthesis of Aristotelian philosophy and Roman law. See J Gordley, ‘Contract Law in the Aristotelian Tradition’ in P Benson (ed), *The Theory of Contract Law: New Essays* (CUP 2001) 265. The idea of contract and contractual obligations can already be seen in late Mesopotamian law, see eg P Barmash, *The Laws of Hammurabi: At the Confluence of Royal and Scribal Traditions* (OUP 2020).

113 Siems (n 13) 182.

114 See Glenn (n 50) 221–6.

115 Zweigert and Kötz (n 34) 40.

116 HC Gutteridge, *Comparative Law* (CUP 1946) 32 (also mentions race and politics).

117 Michaels, ‘On the Comparability of Religious Law’ (n 61) 39 crystallises this by saying that ‘The content of religious law cannot be reduced to the content of holy books’.

118 See G Yurdakul and others, ‘Contested Authorities over Life Politics’ (2019) 18 Comparitive Sociology 706 (compares Germany, Turkey and Israel).

119 Doe (n 31) 388.

120 ‘Religious written law does not stem from formal legal speech acts issued by human legislators and judges. Religious law is not positive but revealed’, K Tuori, *Properties of Law* (CUP 2021) 231. For example, ‘Hindu law is considered to be divine law, a revealed law’, P Diwan and P Diwan, *Hindu Law* (Wadhwa Company 1995) 55 (besides ancient sources, modern sources, including precedents and legislation, are regarded as sources of Hindu law, ibid 84–8).

121 Michaels, ‘On the Comparability of Religious Law’ (n 61) 38.

122 An important illustration is the *Statement of Principles of Christian Law* (2016). For a more detailed discussion, see M Hill and N Doe, ‘Principles of Christian Law’ (2017) 19 Ecc LJ 138.

123 This scholarship relies on the assumption of similarity, which is a key part of so-called functional comparative law. See N Doe, *Christian Law: Contemporary Principles* (CUP 2013) (‘despite the different doctrinal or confessional postures of these separate Christian traditions, there are profound similarities between their contemporary regulatory instruments’ 1); Zweigert and Kötz (n 34) 34–40.

124 The Constitution of the Islamic Republic of Iran 1979, as amended in 1989. See art 2, which defines Iran as an Islamic Republic with a system based on Islam.

125 The Hindu Marriage Act (Act 25 of 1955), 18 May 1955.

126 J Witte Jr and FS Alexander, ‘Introduction’ in J Witte Jr and FS Alexander (eds), *Christianity and Law* (CUP 2008) 1, 1. ‘The meeting of law and religion is … an instance of cross-cultural interaction, and, as such, endlessly unstable and provocative’, BL Berger, *Law’s Religion: Religious Difference and the Claims of Constitutionalism* (Toronto UP 2015) 18.

127 cf Whitman (n 11) 752.

128 See eg D Bradley, ‘Comparative Law, Family Law and Common Law’ (2003) 23 OJLS 127 (compares American and British family law policies). See also Monateri (n 58) 7 (‘family law, where religious customs, historical traditions, and a certain degree of patriarchal attitudes could play a great role in differentiating one legal system from others’).

129 See J Husa, ‘The Future of Legal Families’ in *Oxford Handbook Topics in Law* (OUP 2016) <https://doi.org/10.1093/oxfordhb/9780199935352.013.26> accessed 13 February 2023.

130 cf A Vereshchagin, ‘An Interview with William Twining’ (2021) 16 J Comp L 445, 453.

131 cf Berman (n 9) 748–9.

